# Strigolactone versus gibberellin signaling: reemerging concepts?

**DOI:** 10.1007/s00425-016-2478-6

**Published:** 2016-02-22

**Authors:** Eva-Sophie Wallner, Vadir López-Salmerón, Thomas Greb

**Affiliations:** Centre for Organismal Studies (COS), Heidelberg University, Im Neuenheimer Feld 230, 69120 Heidelberg, Germany

**Keywords:** D53/SMXL, Hormonal signaling, Long-distance communication, SCF complex

## Abstract

**In this review, we compare knowledge about the recently discovered strigolactone signaling pathway and the well established gibberellin signaling pathway to identify gaps of knowledge and putative research directions in strigolactone biology.**

Communication between and inside cells is integral for the vitality of living organisms. Hormonal signaling cascades form a large part of this communication and an understanding of both their complexity and interactive nature is only beginning to emerge. In plants, the strigolactone (SL) signaling pathway is the most recent addition to the classically acting group of hormones and, although fundamental insights have been made, knowledge about the nature and impact of SL signaling is still cursory. This narrow understanding is in spite of the fact that SLs influence a specific spectrum of processes, which includes shoot branching and root system architecture in response, partly, to environmental stimuli. This makes these hormones ideal tools for understanding the coordination of plant growth processes, mechanisms of long-distance communication and developmental plasticity. Here, we summarize current knowledge about SL signaling and employ the well-characterized gibberellin (GA) signaling pathway as a scaffold to highlight emerging features as well as gaps in our knowledge in this context. GA signaling is particularly suitable for this comparison because both signaling cascades share key features of hormone perception and of immediate downstream events. Therefore, our comparative view demonstrates the possible level of complexity and regulatory interfaces of SL signaling.

## Introduction

SLs have a long research history in the context of interactions between plants and other organisms. They were identified in 1966 as plant-derived molecules used by parasitic plants to interact with their hosts (Cook et al. 1966). Further emphasizing their importance for biotic interactions, the role of SLs in the establishment of symbioses between plants and arbuscular mycorrhizal fungi was revealed in 2005 (Akiyama et al. 2005). Only in 2008 were SLs recognized as endogenous phytohormones when their role as decisive hormones regulating plant architecture was uncovered (Gomez-Roldan et al. 2008; Umehara et al. 2008). Since then, research on SL signaling mechanisms has revealed surprising parallels to other hormone signaling cascades, with the most similar being mechanisms of GA perception. Due to the instructive nature of comparative approaches, we relate in this review GA and SL signaling in order to accentuate emerging similarities and differences between the two pathways. Due to the striking parallels between both signaling cascades, we hope that this approach will be helpful for understanding the biological role of SL signaling during plant growth. For example, the presence of different bioactive GAs or the parallel effects of GA on transcription and subcellular localization of proteins demonstrates the complexity of molecular events that should be considered for a comprehensive understanding of a hormonal signaling cascade.

It is important to note, however, that there is no reason to think that SL signaling is more entangled with GA signaling than with other hormonal signaling pathways. Indeed, the interaction between auxin and SL signaling has a long history of research (Waldie et al. 2014; Brewer et al. 2009, 2015; Domagalska and Leyser 2011). Furthermore, the concept that nuclear hormone receptors, inducing the degradation of signaling repressors, extensively discussed in this review, is not restricted to GA and SL signaling but also found in jasmonic acid and auxin signaling cascades (Larrieu and Vernoux 2015). However, for the sake of conciseness we focus on the GA-SL comparison in order to guide the potential routes of SL research and demonstrate gaps in current knowledge. For the same reason, we do not focus on mechanisms of GA or SL biosynthesis, although this is an essential level of regulation, as this has been recently presented in excellent and comprehensive overviews (Seto and Yamaguchi 2014; Hedden and Thomas 2012).

## Similar but different—families of related molecules

**Fig. 1:**
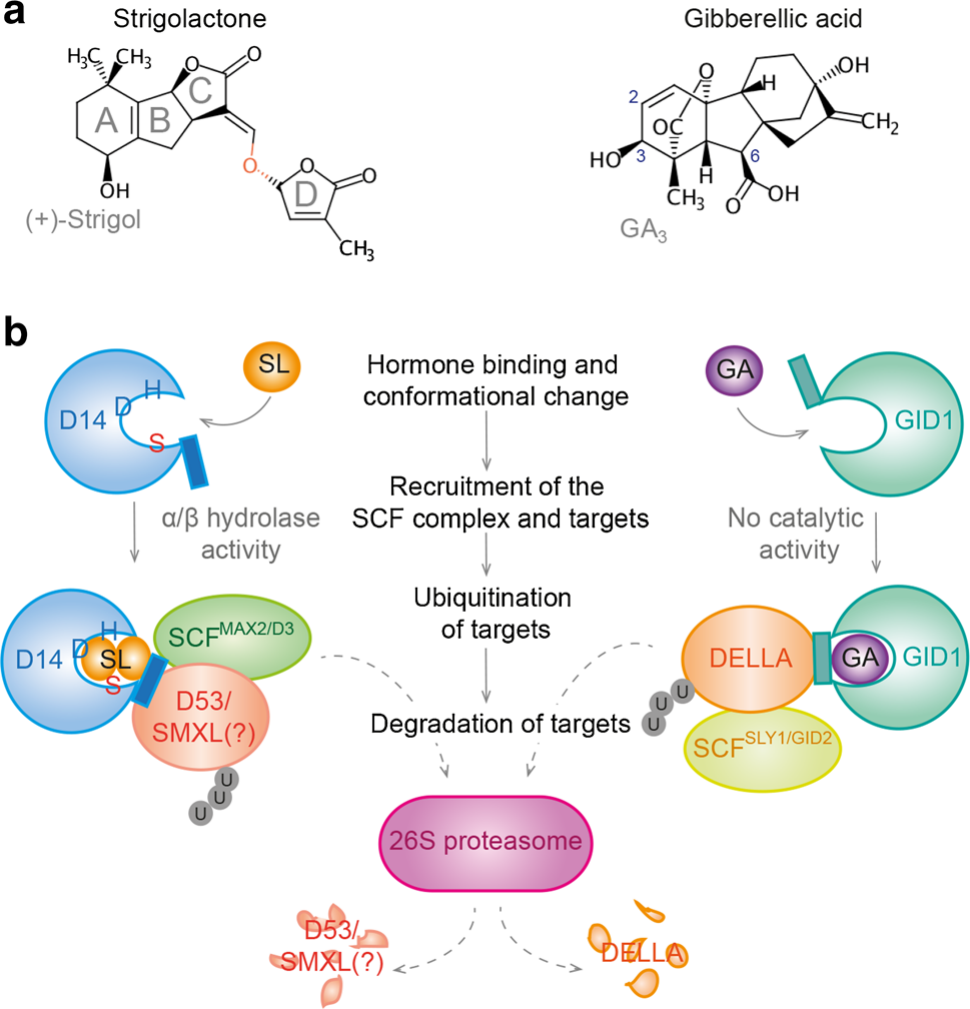
Similarities between SL and GA perception. **a** Molecular structures of SL and GA are exemplified by (+)-5-Deoxystrigol and GA_3_, respectively. The ABC scaffold of SL is connected to ring D by an enol ether bridge (indicated in *orange*). **b** A schematic comparison between SL- and GA-signaling is shown. Unlike GID1, the *α*/*β*-hydrolase D14 preserved its catalytic activity. Bound SL is hydrolyzed through a nucleophilic attack by Ser147 (visualized in *orange*) at the enol ether bridge. Marvin was used for drawing, displaying and characterizing chemical structures, substructures and reactions, Marvin Beans (15.9.28.0), 2015, ChemAxon (http://www.chemaxon.com). Abbreviations, see main text

More than 100 different GAs have been isolated from vascular plants (MacMillan 2001) from which gibberellin A_1_ (GA_1_), GA_3_, GA_4_, GA_5_, GA_6_ and GA_7_ are biologically active. These GAs show different affinities to their receptors (Ueguchi-Tanaka et al. 2005, 2007; Nakajima et al. 2006) and their occurrence and abundance varies between different plant species (MacMillan 2001). For example, whereas GA_1_ is the most widespread gibberellin among species, GA_4_ is the most abundant and relevant bioactive GA in *Arabidopsis* (Eriksson et al. 2006; Talon et al. 1990). The structural requirements for a bioactive GA are clearly defined. These diterpenoid acids must possess a carboxyl group at position C6, a hydroxyl group at position C3 in *β*-orientation and a *γ*-lactone ring. Furthermore, they must not be hydroxylated at position C2, since hydroxylation at this position is critical for inactivation of GA *in planta* (Ueguchi-Tanaka and Matsuoka 2010) (Fig. 1). The stability of different GAs is also important to consider. GA_3_, for instance, shows a lower affinity than GA_4_ to its receptor GIBBERELLIN-INSENSITIVE DWARF1 (GID1) but a higher bioactivity. This is presumably due to increased GA_3_ stability caused by a double bond at the C2 position (Ueguchi-Tanaka et al. 2005).

Although identification of SLs is technically very challenging, around 20 naturally occurring SLs have been described so far (Zwanenburg and Pospisil 2013; Ueno et al. 2014). They all share an ABC scaffold consisting of three carbon rings attached to a butenolide (ring D) by an enol ether bridge (Fig. 1) (Zwanenburg et al. 2015; Xie and Yoneyama 2010). The enol ether bridge determines the bioactivity of SLs, since hydrolytic cleavage between ring C and D is crucial for SL perception and specificity (Zwanenburg et al. 2013, 2015; Mangnus and Zwanenburg 1992). The importance of the CD rings becomes obvious by the finding that an additional methyl group on ring D can significantly decrease the molecule’s ability to induce parasitic seed germination (Zwanenburg et al. 2013). Depending on the stereochemistry of the BC junction, SLs fall into the strigol and orobanchol classes, which show an opposing C-ring orientation determining functional specificity (Zwanenburg et al. 2015; Zhang et al. 2014; Scaffidi et al. 2014). 5-Deoxystrigol (5DS) and 4-deoxyorobanchol (4DS) are most likely the parent molecules that are converted into both classes, respectively, with overlapping but not identical biological activities (Zhang et al. 2014; Zwanenburg et al. 2015; Scaffidi et al. 2014). For instance, members of the strigol class most effectively stimulate germination of the parasitic weed *Striga hermonthica,* whereas orobanchol derivatives show the highest activity in stimulating mycorrhizal hyphal branching (Nomura et al. 2013; Akiyama et al. 2010). Beside these canonical SLs, a major role of non-canonical SLs, like methyl carlactonate, has been discussed especially for *Arabidopsis* (Abe et al. 2014; Zhang et al. 2014).

It is important to note that, although the bioactivity of individual SLs and in vitro receptor binding was shown in some cases (see below), the identification of the active forms *in planta* is a challenging enterprise. This is, in part, because plants may quickly convert applied compounds. A deeper understanding of the SL biosynthetic pathway and analysis of respective mutants will be essential to clarify which features are crucial for bio-availability of naturally occurring SLs (Seto and Yamaguchi 2014). For example, in addition to 2*β*-hydroxylation, bioactive GAs are also inactivated by methylation (Varbanova et al. 2007) and epoxidation of the C-16,17 double bond (Zhu et al. 2006). GA-methyl transferase activity mediated by GIBBERELLIN METHYLTRANSFERASE1 (GAMT1) and GAMT2 appears to be restricted to developing seeds (Varbanova et al. 2007; Nir et al. 2014) whereas 16,17-epoxidation has only been demonstrated in rice (Zhu et al. 2006). In the case of SLs it has not been determined if there are essential regulatory modulations of bioactive SL molecules.

Due to the high variability and specificity within the SL family, artificially produced SL analogs of simplified structure have to be used cautiously (Conn et al. 2015; Zwanenburg et al. 2015). Plants do not produce these analogs, which may, therefore, act very differently from endogenous SLs. For instance, the synthetic and broadly used SL analog GR24 consists of a racemic mixture of natural strigol-like GR24^5DS^ as well as its unnatural enantiomer GR24^ent−5DS^ (Scaffidi et al. 2014; Conn et al. 2015). The natural GR24^5DS^ is most active in repressing SL-dependent shoot branching, whereas GR24^ent−5DS^ preferentially activates the karrikin (KAR)-dependent pathway inducing germination after wildfires (Conn et al. 2015; Umehara et al. 2015; Waters et al. 2014) and important for recruiting arbuscular mycorrhizal fungi in rice (Gutjahr et al. 2015). Therefore, the effects observed after GR24 application are not necessarily natural SL responses.

It has not been reported that different bioactive GAs trigger different responses (Nakajima et al. 2006). All GID1 family members display a similar profile of binding affinities (Nakajima et al. 2006). This is interesting, as triggering specific subsets of downstream responses by different GAs could provide an advantage by providing regulatory flexibility. However, GAs are not only produced by plants but also by fungal pathogens to manipulate plant growth (Bömke and Tudzynski 2009). Prevention of sophisticated growth manipulation by pathogens may be a reason for this lack of signaling complexity among GA molecules. Although KAR receptors may sense fungus-derived signals (see below) (Gutjahr et al. 2015), there is no indication that non-plant pathogens produce SLs. The more complex set of SL-related molecules may be important for the recruitment of host- and/or growth stage-specific sets of symbiotic fungi (Gutjahr 2014) on the one side and the avoidance of parasitic plants (Cardoso et al. 2011) on the other side. Therefore, a spectrum of different SLs with slightly different activity is likely to be under positive selection (Akiyama et al. 2010; Nomura et al. 2013). The presence of canonical SLs in rice, which hosts mycorrhizal fungi, and their apparent absence in the non-host plant *Arabidopsis* (Abe et al. 2014) may be an example for species-specific adaptation.

## The importance of hormone distribution

GAs move over long distances (Ragni et al. 2011; Proebsting et al. 1992) and recently it was suggested that GA_12_, the precursor of bioactive GAs, is the main form traveling along the vasculature (Regnault et al. 2015). Importantly, the finding that fluorescently labelled and bioactive GAs accumulate particularly in the root endodermis suggests that differential accumulation of GAs in plants occurs (Shani et al. 2013). The endodermis is also the most potent tissue for influencing GA-dependent root elongation (Ubeda-Tomas et al. 2008) and a site for GA production (Zhang et al. 2011). Overall, the fundamental role of spatial regulation of hormone levels and signaling is an emerging picture in many contexts (Savaldi-Goldstein et al. 2007; Iyer-Pascuzzi et al. 2011) and is especially established for auxins (Adamowski and Friml 2015).

The spatial distribution of SLs has not been revealed with high resolution; but novel fluorescent and bioactive SL analogs may provide an angle for filling this gap of knowledge (Prandi et al. 2013; Rasmussen et al. 2013b; Artuso et al. 2015; Fridlender et al. 2015). The expression of SL biosynthesis genes is usually highest in roots and partially associated with vascular tissues (Booker et al. 2005; Kohlen et al. 2012). Indeed, SL-like bioactivity has been found in the *Arabidopsis* xylem sap (Kohlen et al. 2011). Although an important role of canonical SLs in *Arabidopsis* was questioned in later studies (Abe et al. 2014), orobanchol was identified directly in the tomato and *Arabidopsis* xylem sap, pointing out a possibility for long-distance movement (Kohlen et al. 2011,  2012). In any case, movement of SLs—or their precursors—is able to completely suppress effects of SL-deficiency in grafting experiments with a preferred directionality for traveling from roots to shoots (Foo and Davies 2011; Turnbull et al. 2002; Booker et al. 2005). The low pH usually found in the xylem sap (Jia and Davies 2007) would support SL stability (Zwanenburg et al. 2015). Candidates for moving long distances are carlactonoic acid and orobanchol, the suggested products of MORE AXILLARY GROWTH1 (MAX1)-like enzymes, which catalyze the last step in the SL biosynthetic chain (Abe et al. 2014; Zhang et al. 2014; Booker et al. 2005). Consequently, the diverse regulatory roles of SLs, such as inhibiting shoot branching, promoting cambium activity and regulating root growth, partly in response to environmental cues (Umehara et al. 2010), would provide a means for coordinating plant growth processes in a systemic manner (Agustí et al. 2011; Gomez-Roldan et al. 2008; Rasmussen et al. 2013a; Umehara et al. 2010). However, the relevance of hormone movement under natural conditions is difficult to demonstrate without a possibility to manipulate this movement in a very specific manner. The lack of knowledge on how GA travels through the plant has hampered research in this direction so far. The discovery that the ABC transporter PLEIOTROPIC DRUG RESISTANCE1 (PDR1) from petunia (*Petunia axillaris*) is involved in SL secretion into the rhizosphere (Kretzschmar et al. 2012) and localizes polarly in plasma membranes (Sasse et al. 2015) may provide a novel avenue in this context. Thus, in addition to a passive long-distance movement, mechanisms for establishing local SL maxima may exist, which are relevant for local and cell type-specific responses.

## The conversion of enzymes into receptors

The most striking analogy between GA and SL signaling is the mechanism of perception. The nuclear-localized and soluble protein GID1 is a catalytically inactive *α*/*β*-hydrolase identified in rice, which binds bioactive GAs (Ueguchi-Tanaka et al. 2005; Shimada et al. 2008). In comparison to rice, which possesses only one *GID1* gene, there are three redundant *GID1* genes (*GID1a,*−*b* and −*c*) in *Arabidopsis* (Nakajima et al. 2006; Griffiths et al. 2006). Single mutants show only mild phenotypic alterations, but the *gid1a/b/c* triple mutant displays an extremely dwarfed growth habit and complete GA insensitivity (Griffiths et al. 2006; Ueguchi-Tanaka et al. 2005). This indicates that these proteins are the only GA receptors. The crystal structure of the GID1 receptor has helped to understand its function and the structural requirements that define a bioactive GA (Shimada et al. 2008). GA binding triggers a conformational change in the GID1 protein. This change promotes direct interaction of the GA-GID1 complex with DELLA proteins acting as transcriptional regulators (Harberd et al. 2009; Sun 2011). Formation of the GA-GID-DELLA ternary complex, in turn, recruits the SCF^SLY1^ (SKP1, CULLIN, F-box and RBX1 RING-domain) ubiquitin ligase (E3) complex via the F-box protein SLEEPY1 (SLY1), which provides substrate specificity to the complex (Dill et al. 2004) (Fig. 1).

As described below, physical contact of DELLA proteins with the SCF^SLY1^ complex results in their ubiquitination and degradation by the 26S proteasome (Harberd et al. 2009; Dill et al. 2004). Removal of the nuclear DELLA proteins results in massive changes in gene expression and, among other things, culminates in cell elongation (Harberd et al. 2009). In this respect, it is remarkable that *sly1* mutants (or *gid2* mutants in rice) show much milder phenotypic alterations than *gid1a/b/c* mutants do, although they accumulate comparable or even higher levels of DELLAs. Intriguingly, overexpression of the GID1 receptor suppresses these alterations (Ariizumi et al. 2008; Ueguchi-Tanaka et al. 2008). Thus, GID1 proteins may also play a GA-independent role in modulating DELLA activity, by sequestering these repressors into an inactive complex (Ariizumi et al. 2008; Ueguchi-Tanaka et al. 2008; Hauvermale et al. 2014).

In analogy to GA perception, substantial evidence has been provided that SLs bind to the *α*/*β* hydrolase DWARF14/DECREASED IN APICAL DOMINANCE2 (D14/DAD2) (Kagiyama et al. 2013; Zhou et al. 2013; Hamiaux et al. 2012). The binding pocket of D14/DAD2 contains the catalytic tirade Ser147, Asp268 and His297, which hydrolyzes the enol ether bridge between the C and D ring through a nucleophilic attack by Ser147 (Fig. 1) (Kagiyama et al. 2013; Zhao et al. 2015). Any similar activity has been lost in GID1 due to an amino acid substitution that replaced His by Val (Ueguchi-Tanaka et al. 2005). Because reaction products of D14/DAD2 do not display any biological activity, the decisive step in signal transduction is the conformational change of the D14/DAD2 protein and not the generation of signaling molecules (Hamiaux et al. 2012). D14/DAD2 is homologous to the KAR receptor KARRIKIN INSENSITIVE2 (KAI2). However, structure determination and binding analyses revealed that only D14/DAD2 binds SLs (Guo et al. 2013; Conn et al. 2015; Nakamura et al. 2013; Hamiaux et al. 2012; Toh et al. 2015; Zhao et al. 2015). In fact, it seems as if diversification of SL receptor-like proteins was crucial for the establishment of these distinct signaling cascades (Conn et al. 2015; Waters et al. 2012), a situation not found in the case of GID1. In addition to mediating KAR-dependent seed germination in some species, still unknown endogenous KAI2-binding molecules must exist because *kai2* mutants display also developmental defects (Nelson et al. 2011; Waters et al. 2012). Interestingly, the KAI2 ortholog D14L in rice is essential for the recognition of arbuscular mycorrhizal fungi and the initiation of symbiotic interactions (Gutjahr et al. 2015). Thus, SL/KAR-related molecules do not only act as attractants during biotic interactions but their endogenous perception machinery is also important for recruiting symbiotic organisms. This argues for an intensive SL/KAR-dependent cross talk bridging species boundaries. The existence of a third D14/DAD2-like protein in *Arabidopsis* designated as D14-LIKE2 (DLK2), which does not contribute to SL or KAR responsiveness (Waters et al. 2012), suggests an even more complex situation on this level.

Similar to GID1, D14/DAD2 changes conformation upon SL binding which facilitates the interaction with the F-box protein and SCF complex component DWARF3 (D3). D3 is the rice ortholog to MORE AXILLARY GROWTH2 (MAX2) from *Arabidopsis* which is mainly expressed in vascular tissues (Chevalier et al. 2014; de Saint et al. 2013a, b; Zhou et al. 2013; Jiang et al. 2013; Stirnberg et al. 2007). Binding of D3/MAX2 to D14/DAD2 occurs close to its lid domain (Zhao et al. 2015) (Fig. 1). In comparison to SLY1, which can be partly replaced by the F-box protein SNEEZY (SNE) (Ariizumi et al. 2011), D3/MAX2 is the only F-box protein known to act in SL signaling. In fact, D3/MAX2 plays a key role in both the D14/DAD2 and KAI2-dependent signaling pathways (Waters et al. 2012). Interestingly, an exclusive role of D3/MAX2 in SL/KAR-signaling is questioned by the observation that *max2* mutants respond to higher GR24 concentrations (Ruyter-Spira et al. 2011; Agustí et al. 2011) for which the basis still has to be determined. As explained in more detail below, the SCF^D3/MAX2^ E3 ubiquitin ligase complex executes SL-dependent ubiquitination of target proteins, such as DWARF53 (D53) in rice (Jiang et al. 2013). Just as the ubiquitination machinery of GA signaling and its DELLA targets, D3/MAX2, D14 and D53 are nuclear localized (Jiang et al. 2013; Stirnberg et al. 2007; Nakamura et al. 2013), thereby providing a potential link to a direct regulation of gene transcription.

Of note, GA and SL signaling pathways have been suggested to directly interact with each other. Hydrolyzation of SL/GR24 enables D14 to bind not only to D53-like proteins but also SLENDER1 (SLR1), the only DELLA protein found in rice (Nakamura et al. 2013). Thereby, SLs may contribute to GA signaling and suppress bud outgrowth in rice (Nakamura et al. 2013). However, D14-SLR1 binding was only shown indirectly using heterologous expression systems, and there is no physiological or genetic evidence that both pathways intertwine functionally. Instead, there are indications favoring an independent action. SL signaling promotes internode elongation in peas by increasing cell number, not by stimulating cell elongation as primarily done by GA (de Saint et al. 2013b). Furthermore, GA, but not GR24, application destabilizes DELLA proteins, GA responsiveness is not affected in SL signaling mutants and their dwarfism is not correlated with reduced GA levels (de Saint et al. 2013b). Further supporting an independent action, SL signaling acts antagonistically rather than in concert with GA signaling in the regulation of shoot branching in the woody plant *Jatropha curcas* (Ni et al. 2015).

## Direct targets of signaling—the reemerging motif of repressing repressors

As mentioned, binding of GA or SLs to their respective receptor complexes leads to the 26S proteasome-dependent degradation of two distinct groups of signaling repressors: DELLA proteins in the case of GA and D53-like proteins in the case of SLs (Jiang et al. 2013; Zhou et al. 2013). DELLA proteins belong to the larger family of GRAS transcriptional regulators, which seem to have diversified to allow the integration of GA signaling into transcriptional regulation. DELLA proteins are named after their *N*-terminally conserved amino acid sequence (D–E–L–L–A) essential for binding to GID1 (Schwechheimer and Willige 2009; Wang and Deng 2011). In *Arabidopsis*, the GRAS proteins GA-INSENSITIVE (GAI), REPRESSOR OF GA1-3 (RGA), RGA-LIKE1 (RGL1), RGL2 and RGL3 carry such a domain (Dill et al. 2004). Although partially redundant, the five DELLA proteins display a certain functional specialization, such as the regulation of germination, stem elongation, leaf expansion, apical dominance or floral development (Dill et al. 2004; Wang and Deng 2011). While this specialization appears to result rather from their distinct expression patterns than from differences in protein properties (Gallego-Bartolome et al. 2010), there is an indication that there are differences in GA-induced degradation kinetics among the DELLA proteins (Wang et al. 2009) although more accurate studies are required to confirm these differences. Interestingly, in contrast to SLY1 which targets all DELLAs equally, SNE preferentially targets RGA and GAI, thus providing the possibility for a differential regulation of DELLA protein abundance on the level of the GA perception machinery (Ariizumi et al. 2011).

**Fig. 2:**
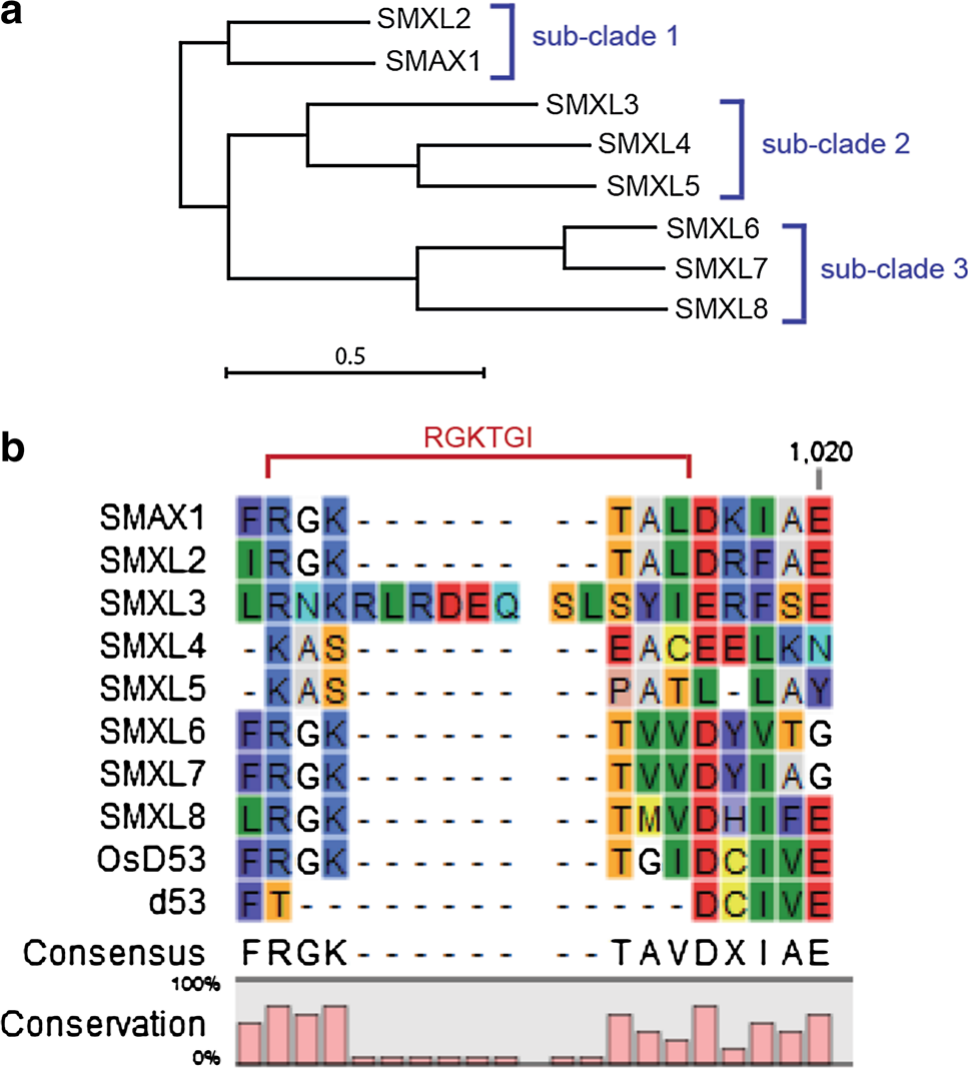
Comparison of D53/SMXL family members. **a** A maximum likelihood phylogenic tree based on an amino acid sequence alignment of the *Arabidopsis* SMXL proteins. The *scale bar* indicates a branch length with 0.5 amino acid substitutions per site. The three putative sub-clades are emphasized by *blue brackets*. CLC Main Workbench 7.6.1 (CLC Bio Qiagen, Denmark). **b** Shown is the motif important for D3-dependent ubiquitination of D53 from rice identified previously (Jiang et al. 2013; Zhou et al. 2013). Aligned are the eight SMXL family members from *Arabidopsis*, the SMXL rice homolog D53 (OsD53) and the mutated d53 protein in which this motif is lost (indicated by a *red bracket*). Note that the RGKTGI motif is not present in members of sub-clade 2. CLC Main Workbench 7.6.1 (CLC Bio Qiagen, Denmark)

The reasonable assumption that SL signaling depends on the proteolysis of a set of repressor proteins was confirmed by the seminal identification of the D53 protein in rice which is nuclear localized and shows weak similarities to Class 1 Hsp100/ClpB proteins (Jiang et al. 2013; Zhou et al. 2013). Reminiscent of the GA-effect on DELLA proteins, D53 interacts with both D3 and D14 in an SL-dependent manner and is subsequently ubiquitinated and degraded (Jiang et al. 2013; Zhou et al. 2013). The *d53* rice mutant carries a dominant-negative allele producing a protein with a deletion of five amino acids (GKTGI) and an amino acid substitution that changes a positively charged Arg into a Thr (Fig. 2). This alteration results in GR24-insensitivity and a dwarfed and bushy phenotype indicative of reduced SL signaling (Jiang et al. 2013; Zhou et al. 2013). Although both D53 and the mutated *d53* protein are able to interact with D14, only D53 undergoes SL-dependent proteolysis (Jiang et al. 2013). This indicates that, unlike the DELLA motif, the RGKTGI sequence is crucial for the D14–D3-complex dependent ubiquitination but not for the interaction with the SL receptor complex. In fact, the part of D53-like proteins that interacts with the D14–D3 complex is still to be determined.

Consistent with the idea that the SL signaling mechanism is conserved across species boundaries, the D53 homologue SUPPRESSOR OF MAX2 1 (SMAX1) was identified in *Arabidopsis* in an elegant forward genetic screen for suppressors of effects of impaired SL/KAR signaling (Stanga et al. 2013). SMAX1 defined the small gene family of SMAX1-LIKE (SMXL) proteins consisting of eight members in *Arabidopsis* (Fig. 2). Similar to the DELLA proteins, differences in specificity and function have been proposed for SMXL family members (Stanga et al. 2013). The *smax1 max2* mutant suppresses hypocotyl and germination defects found in *max2* mutants, but not the typical increase in shoot branching, which is primarily associated with SL-deficiency (Stanga et al. 2013). Because *SMAX1* and *SMXL2*, the two members of the D53/SMXL sub-clade 1, are sufficient for regulating all KAR-dependent responses, a functional separation of the D53/SMXL family into KAR and SL-signaling factors is likely (Stanga et al. 2013, 2016; Waters et al. 2014). Consistent with this idea, triple mutants lacking the activity of the clade 3-family members, *SMXL6*, *SMXL7* and *SMXL8*, fully suppress all SL-related growth alterations caused by *MAX2*-deficiency (Wang et al. 2015; Soundappan et al. 2015). As with D53 in rice, the nuclear-localized SMXL6, SMXL7 and SMXL8 proteins are ubiquitinated and degraded upon the addition of GR24. Likewise, they interact with D3/MAX2 and D14 proteins (Wang et al. 2015; Soundappan et al. 2015). Interestingly, artificial miRNAs (amiRNAs) targeting *SMXL6, SMXL7* and *SMXL8* transcripts suppressed the *max2*-specific increase in shoot branching but not amiRNAs targeting the sub-clade 2 members *SMXL4* and *SMXL5* (Soundappan et al. 2015). Although the third sub-clade member, *SMXL3*, was not repressed in *smxl45*-*ami max2* plants, these results are in agreement with the idea that members of clade 3 mediate SL signaling while other SMXL proteins fulfill different functions (Wang et al. 2015; Soundappan et al. 2015).

Supporting this assumption, the RGKTGI motif identified to be important for SL/KAR-dependent degradation (Jiang et al. 2013; Zhou et al. 2013; Soundappan et al. 2015) is not conserved in SMXL proteins belonging to clade 2 (Fig. 2). This opens up the possibility that members of this clade are SL/KAR-independent reminiscent to the situation in the GRAS family from which only a subset is GA-dependent. However, expression patterns of different family members are very diverse (Stanga et al. 2013; Soundappan et al. 2015) making it possible that, when compared to the DELLAs, the emerging differences in function are simply due to different sites of action. Looking again at GA signaling, posttranslational modification is important for DELLA activity. *O*-GlcNAcylation catalyzed by the GlcNAc transferase SPINDLY (SPY) promotes DELLA activity (Silverstone et al. 2007). Moreover, stress-dependent SUMOylation of DELLAs allows stable binding to GID1 independently from GA, resulting in reduced degradation of non-SUMOylated DELLAs and, therefore, decreased GA-sensitivity (Conti et al. 2014). Thus, presence or absence of SMXL proteins may not be the only critical aspect for determining the level of SL signaling in particular contexts.

## The complexity of downstream processes

DELLAs, similarly to D53/SMXL proteins, do not contain a canonical DNA binding domain. However, DELLAs interact with several groups of transcription factors, thereby, preventing their DNA binding (Xu et al. 2014). Famous examples are the PHYTOCHROME INTERACTING FACTORS (PIFs). GA-dependent DELLA degradation releases these basic helix-loop-helix (bHLH) transcription factors and induces the transcription of genes which are conversely regulated by light through phytochrome-dependent PIF degradation (Huq and Quail 2002; Khanna et al. 2004; de Lucas et al. 2008; Feng et al. 2008). Thus, GA- and light signaling converge on the level of PIF transcription factors, nicely demonstrating how opposing stimuli are integrated on the molecular level. Likewise, DELLAs stimulate jasmonic acid (JA) signaling by titrating away JA ZIM-domain (JAZ) proteins acting as JA signaling repressors (Hou et al. 2010) and dampen brassinosteroid (BR) signaling by binding to the BRASSINAZOLE-RESISTANT1 (BZR1) transcription factor important for BR-dependent gene activation (Gallego-Bartolome et al. 2012; Bai et al. 2012). These findings reveal an astonishing broadness of direct interactive connections between different hormone-dependent transcriptional regulators and underline the necessity for integrative approaches to understand downstream responses.

In addition to interfering with the activity of other transcription factors, evidence for a direct stimulation of transcription has been documented, for example for *SLR1* from rice (Hirano et al. 2012). The mystery of how DELLAs interact with DNA in this context has been elucidated recently by the identification of the DNA-binding INDETERMINATE DOMAIN (IDD) family proteins, which serve as transcriptional scaffolds in *Arabidopsis* (Yoshida et al. 2014). This study shows that IDD proteins are important for GA signaling and bind to both, the promoter of the *SCARECROW-LIKE3* (*SCL3*) gene and to the RGA protein (Yoshida et al. 2014).

Beyond the direct or indirect regulation of transcription, DELLAs also titrate away proteins that move from the nucleus to the cytoplasm upon DELLA degradation to execute their function. In particular, the prefoldin complex (PFD), a co-chaperone required for tubulin folding, translocates after GA-induced DELLA degradation and increases the amount of active tubulin subunits promoting cell expansion (Locascio et al. 2013). Thus, DELLAs act as central hubs for executing GA signaling and integrating various signaling pathways on multiple cellular levels.

The molecular role of D53/SMXL proteins is still obscure. They are large (around 1000 amino acids) providing plenty of opportunities for interactions with other molecules. Indeed, D53/SMXL proteins carry a putative ethylene-responsive element binding factor-associated amphiphilic repression (EAR) domain that can interact with TOPLESS (TPL) (Jiang et al. 2013; Soundappan et al. 2015; Wang et al. 2015). TLP and TLP- RELATED (TRP) proteins are well studied repressors of transcription in plants and were found to specifically interact with transcription factors to regulate many growth processes (Causier et al. 2012). D53, SMAX1, SMXL6, SMXL7 and SMXL8 interact with TLP proteins in heterologous expression systems and in vitro (Jiang et al. 2013; Soundappan et al. 2015; Wang et al. 2015). Although the functional relevance of these interactions remains to be tested, this connection may help identifying downstream targets of SL-signaling and mechanisms of SL-dependent gene regulation.

Interestingly, SL signaling has been proposed to act in parallel to light perception by preventing the E3 ubiquitin-ligase CONSTITUTIVE PHOTOMORPHOGENIC1 (COP1) from entering the nucleus and degrading the light-responsive protein LONG HYPOCOTYL5 (HY5) (Tsuchiya et al. 2010; Jia et al. 2014). HY5 is a bZIP transcription factor antagonizing PIF activity by competing for the same promoter binding sites (Toledo-Ortiz et al. 2014). One of its best-known functions is the inhibition of hypocotyl elongation, which is used as a common readout to determine light- and/or SL/KAR-sensitivity in *Arabidopsis* (Jia et al. 2014; Scaffidi et al. 2013). GR24 suppresses hypocotyl elongation in a light- and *MAX2*-dependent manner (Jia et al. 2014). Moreover, *hy5* and *max2* mutants display an additive effect regarding GR24-insensitivity (Shen et al. 2012). Thus, although the exact molecular mechanism is so far unknown and highly debated, it has been hypothesized that *MAX2* regulates photomorphogenesis (Jia et al. 2014; Waters and Smith 2013; Tsuchiya et al. 2010; Shen et al. 2012). However, as mentioned above, GR24 effects and a role of *MAX2* are not necessarily indicative of a role of SL signaling in mediating the effect of light, as both are not specific for this pathway. Indeed, SL-deficient mutants usually do not display canonical light-related phenotypic alterations in a broad spectrum of species including *Arabidopsis* and pea (Urquhart et al. 2015; Shen et al. 2012). Furthermore, although *hy5* and photoreceptor mutants are hyposensitive against GR24 and KAR treatments with respect to the repression of hypocotyl elongation (Jia et al. 2014; Waters and Smith 2013), molecular responses are not affected (Waters and Smith 2013) suggesting that SL-signaling, as such, is not part of the classic light signaling network.

Apart from being secreted by plant roots and their role in biotic interactions (Xie and Yoneyama 2010), SLs are best known as branching inhibitors (Brewer et al. 2009; Gomez-Roldan et al. 2008). In this case, a negative effect on polar auxin transport by reducing the amount of PIN- FORMED (PIN) auxin exporters in the plasma membrane has been demonstrated (Bennett et al. 2006; Shinohara et al. 2013). Computational modeling supports the idea that limiting auxin transport capacities is a crucial function of SLs in branching control. In this context, SLs enhance competition of branches for auxin transport capacities rather than acting as constitutive inhibitors (Crawford et al. 2010; Shinohara et al. 2013; Prusinkiewicz et al. 2009). In addition, local transcriptional activation of genes influencing branching, such as the TCP transcription factor BRANCHED1 (BRC1), has been described (Braun et al. 2012; Dun et al. 2012). Although the two roles of SL signaling in the regulation of branching has been discussed controversially (Brewer et al. 2015; Waldie et al. 2014), the multitude of direct targets of GA signaling, their parallel mode of action and spatial differences in the signaling process, provides a glimpse of the possible complexity and argues for an integration of different approaches.

## Conclusion

Due to recent fundamental breakthroughs in SL biology research, we expect the unfolding of another complex signaling network in plants soon. In particular, the identification of the D53/SMXL protein family as repressors of SL signaling and direct targets of SL-dependent proteolysis opens up novel avenues to core events in the signaling cascade. Their characterization will be tremendously helpful for integrating the SL pathway into known regulatory networks and for understanding primary effects of SL signaling. Comparisons to other signaling cascades, like GA signaling, are certainly helpful as a first guideline in this regard. Such a comparison demonstrates the degree of complexity possible on the level of transport, perception, and targeted processes and emphasizes experimental pitfalls to be taken into consideration. For example, it will be essential to decipher the roles of the different SLs *in planta* and unwrap their distinct adaptive values. The spatio-temporal dynamics of SL signaling is another interesting aspect for which hardly any information is available. Do all cells have the capacity to sense SLs or is this mainly restricted to vascular tissues? Does sensitivity change over time or in different environments? The identification of events downstream of D53/SMXL proteolysis will certainly provide important insights and tools for addressing these questions. The dissimilarity of D53/SMXL proteins to any other group of known developmental regulators suggests the existence of unique molecular mechanisms and argues for surprising findings in the future.

### *Author contribution statement*

EW, VL and TG wrote the manuscript. All authors read and approved the manuscript.

## Abbrevations

GA: Gibberellin
KAR: Karrikin
SL: Strigolactone
